# P-300. Characteristics associated with HIV pre-exposure prophylaxis persistence among men who have sex with men in the United States: results from the American Men’s Internet Survey (AMIS) 2023-24

**DOI:** 10.1093/ofid/ofaf695.520

**Published:** 2026-01-11

**Authors:** Duygu Islek, Travis Sanchez, Stefan Baral, Supriya Sarkar, Cristian Acero, Jennifer L Glick, Jeb Jones, Iaah Lucas, Leigh Ragone, Mariah Valentine-Graves, Michael Smith, Vani Vannappagari

**Affiliations:** Emory University, Rollins School of Public Health, Atlanta, Georgia; Emory University, Atlanta, GA; Johns Hopkins University, Baltimore, Maryland; ViiV Healthcare, Baltimore, Maryland; Emory University, Rollins School of Public Health, Atlanta, Georgia; Louisiana State University Health Sciences Center, Community Health Science & Policy, New Orleans, Louisiana; Emory University, Rollins School of Public Health, Atlanta, Georgia; Emory University, Rollins School of Public Health, Atlanta, Georgia; ViiV Healthcare, Baltimore, Maryland; Emory University, Rollins School of Public Health, Atlanta, Georgia; Emory University, Rollins School of Public Health, Atlanta, Georgia; ViiV Healthcare, Baltimore, Maryland

## Abstract

**Background:**

Men who have sex with men (MSM) have a high likelihood of HIV acquisition, yet information about pre-exposure prophylaxis (PrEP) persistence among MSM remains scarce. We examined PrEP persistence and associated characteristics among a nationwide sample of US MSM.

**Table 1.**
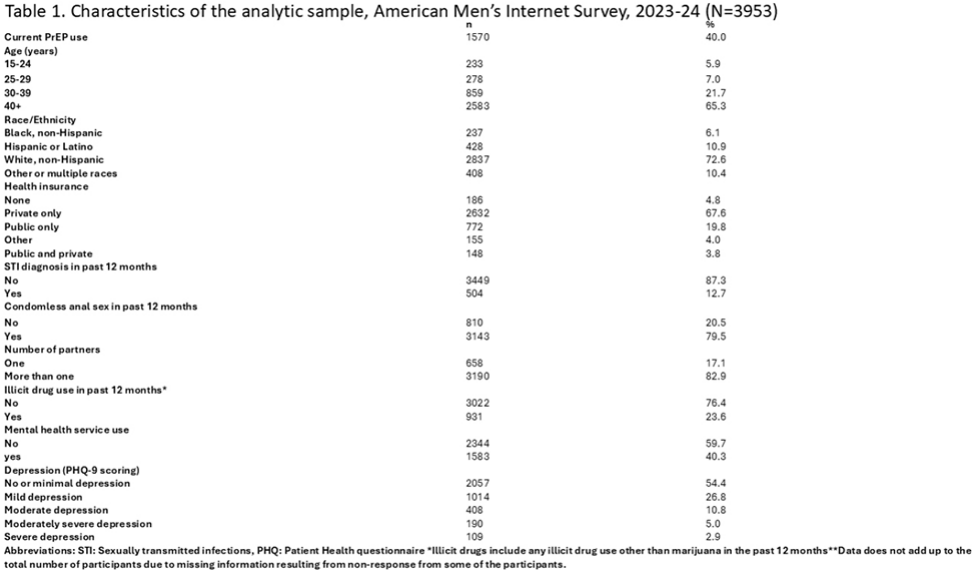
Characteristics of the analytic sample, American Men’s Internet Survey, 2023-24 (N=3953)

**Table 2.**
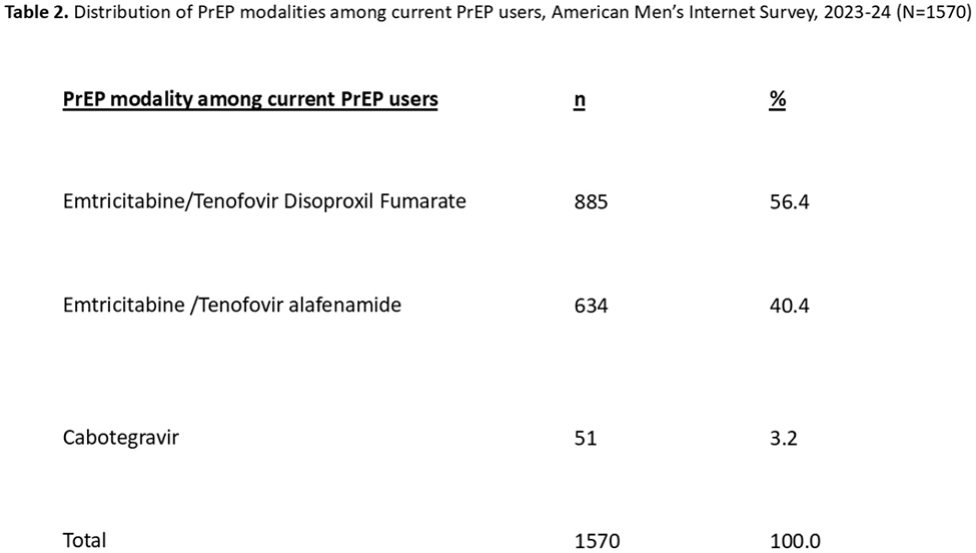
Distribution of PrEP modalities among current PrEP users, American Men’s Internet Survey, 2023-24 (N=1570)

**Methods:**

Sexually active MSM age 15+ were recruited online between December 2023-August 2024. MSM not living with HIV were asked about PrEP use, modality, persistence, and switching. PrEP persistence was described as continuous use of any type of PrEP for the past 12 months or more. Poisson regression models were used to examine associations between various sociodemographic and behavioral characteristics, mental health service use in past 12 months, depression, and PrEP persistence. Depression was defined using Patient Health Questionnaire-9.

**Table 3.**
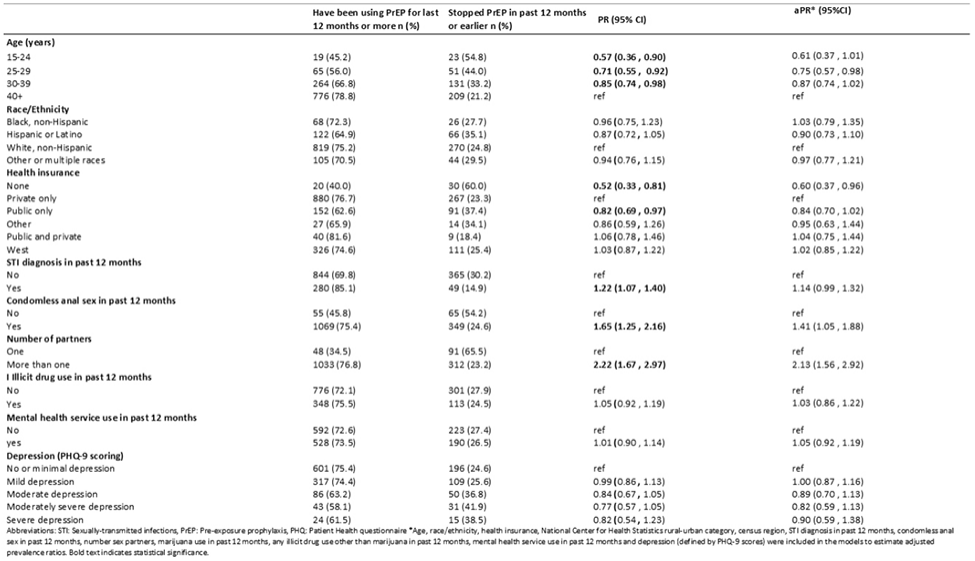
Association between sociodemographic and behavioral characteristics with pre-exposure prophylaxis persistence among men who have sex with men, AMIS, 2023-24

**Results:**

Among 3953 MSM (Table 1), 40% (1570/3953) were currently using PrEP (Table 2) and 72% of those (1124/1562) had been using PrEP more than 12 months. Among participants who have been using PrEP for less than 12 months, 16% (70/438) had switched to another PrEP medication. Emtricitabine/Tenofovir Disoproxil Fumarate was the medication most frequently switched to by participants (58%), followed by Emtricitabine /Tenofovir alafenamide (38%), and Cabotegravir (4%). In multivariable modelling, MSM aged ’25-29’ and MSM with no health insurance were less likely to persistently use PrEP for at least 12 months, compared to MSM 40+ and MSM with private insurance, respectively. MSM who had anal sex and who had more than one sexual partner in the past 12 months were more likely to persistently use PrEP for at least 12 months. There was no association of mental health service use, illicit drug use, or depression with PrEP persistence (Table 3).

**Conclusion:**

A significant proportion of PrEP users maintain continuous use for over 12 months, with age, health insurance status, and sexual behaviors playing crucial roles in persistence. These findings underscore the importance of targeting interventions to younger MSM and those without health insurance to improve long-term PrEP use. These results can inform strategies to enhance PrEP persistence among MSM, such as uptake of novel modalities like long-acting PrEP, ultimately contributing to more effective HIV prevention.

**Disclosures:**

Travis Sanchez, DVM, MPH, ViiV Healthcare, Inc.: Grant/Research Support Supriya Sarkar, PhD, MPH, ViiV Healthcare: Stocks/Bonds (Public Company) Leigh Ragone, MS, GSK: Stocks/Bonds (Private Company)|ViiV Healthcare: Employee Vani Vannappagari, MBBS, MPH, PhD, ViiV Healthcare: Full time Employee of ViiV Healthcare and owns GSK stock|ViiV Healthcare: Stocks/Bonds (Public Company)

